# Supplementary material for: Microsatellite loci development and population genetics in Neotropical fish Curimata mivartii (Characiformes: Curimatidae)
Source: PeerJ. 2018 Nov 13;6:e5959. doi: 10.7717/peerj.5959 (PMC6238776; doi:10.7717/peerj.5959)
Supplement: Supplemental Information 2 [file peerj-06-5959-s002.pdf]

Raw sequence reads, longitude and some identification parameters of 27 microsatellite loci selected for *Curimata mivartii*. Forward and reverse primers are indicated respectively by dark and light gray. Microsatellite motifs are highligthed in green.

Locus Cm01, motif ATCT (reverse complement: AGAT)

>IH3P4PI01A63T5 length=239 xy=0373_3483 region=1 run=R_2013_10_22_16_44_39_

GTGTGCTATTTGGGTTAATGTATAGGTACAGATTGGTGCATTGTTTTATTACTG
ATAGATAGATAGATAGATAGATAGATAGATAGATAGATAGATAGATAGATAGATAG
ATAGATAGATAGATAGATAGATAGATAGATAGATAGATAGATAGATAGATAGATAG
ATAGATAGATAGATAATCTTTATACTTTACATGCTCAAAGTTGCATGTTCAAAG
TTAGCATCATATGAGTCACCAAGCAAG

Locus Cm02, motif ATCT (reverse complement: AGAT)

>IH3P4PI01D5MZF length=388 xy=1587_1289 region=1 run=R_2013_10_22_16_44_39_

GTTTATTTATTCCTTGTTCCTTGCATAGCACCCACAGCCACTCCATGTTTACTTC
AGGAAATGCACTGTTTTAGATAGATAGATAGATAGATAGATAGATAGATAGAT
AGATAGATAGATAGATAGATAGATAGATAGATAGATAGATAGATAGATAGAT
AGATAGATAGATAGATAGATATAGCAATATAGCATATATCTACTGTTCTAGAG
ATCCCATTCATAAGGACACTACCTGCTGATGAACAGGCCACCTTTCTGATCTGA
ATTGATGCTCTGAGAAGTGATCCAGGTCATCTTATGTCATACATATATCCTACG
GAGTAGATGTGGTTAATCAAATGAGACAGAAATGAATTAACCCCTAATGGATC
CGAGCCATATCAGT

Locus Cm03, motif ATCT

>IH3P4PI01DYORW length=506 xy=1508_0602 region=1
run=R_2013_10_22_16_44_39_

ATACTAATAGTTATGTTTATACTGATTGGATATTAATTGACCTCATTACATTGTA
TGCAACAGCTGAAACACAACAAATCTGTTTTATATGCTTATGTAAATATAGATC
TATCTATCTATCTATCTATCTATCTATCTATCTATCTATCTATCTATCTATCTATC
TATCTATCTATCTATCTATCTATCTATCTAATAGTGCTGCTATAATACAATTACA
AATTCCCACCACGAGGTCTCCAAATCCCTAAAACTTACACAGTGCAGCTTTAAT
GAAATACTAAAATGGCTATGCAAGTCTGAAGTAAGCTGGGGAAAAATTTTACT
GAAAACTGAAATTATTTTTTAAAAGCCAGAGCAAACAAAACGTGTGATCTATT
TATGTATTTCTGGATGCATTAACTACTCATACTAATCACTTTATATTTTTACTGC
CTGGTAGAGGAGTCATCATGGGGTAGTTGATAGCATTATATAACTTGTTCCTAT
CTTGTACTATTTTTAAG

Locus Cm05, motif ATCT

>IH3P4PI01E1UIW length=486 xy=1954_0822 region=1 run=R_2013_10_22_16_44_39_

ACTATTAACACTTTACTGTACATCCTGTACGTTTACACTCCTGGACACTATTAAC
ACTTTACTGTACATCCTGCACGTTTACACTCCTGGACACTATTTAACACTTTACT
GTACATCCTGTATGTTGCTCTTCTGGACACTATTAACACTTTTCTTAAATATAAC
TCTTTACACACTGCTATCTATCTATCTATCTATCTATCTATCTATCTATCTATCTA
TCTATCTATCTATCTATCTATCTTTAGTAGAAAACAGAAGTCAGGTAAAATGTA
CATAAACAGGTACAGATTTCATTTATAGCTCCCAGGTTTTAAACAATGTTGTGT
TCACACGTCTAGATGATGTCATTCAGGTGCACAGACAGGAGGAGCTCCATCTT
AAGCTTACACCATAAAACTAAGACACATTCACATACATATTCGACACGAGGCT
AGCTAAACCACCAACTCATCATAATTACACATAGTATTGACTGCACTGTGT

Locus Cm07, motif ATCT

>IH3P4PI01CJ6ML length=645 xy=0932_3579 region=1 run=R_2013_10_22_16_44_39_

AACTGTTTGAGTTCTGAGTTTTGGCATAATTTAGAGCAATAAATGATGTTTGTT
ATATTTTAGTTATTTCTATCTATCTATCTATCTATCTATCTATCTATCTATCTATC
TATCTATCTATGTGTTTGTCTGTCTGAGCTTTGGTGATAAACACATTATCAGAGT
GGCGATTACAGGACAGATCAAATGAACTTTGACGCAGAATGCCAGACACACAG
GAGCCACGACACAAACATTAAACCCTAATGAGCACGCAGTAGACTAATAACGT
GACCATTATGCCAAACGAACTATTTCCAGACACAGATGTTTAAATGTGGTTACA
AGTCAACTTTAATGCAAACTCATCTCTAAACAGCACACAGGCTGCTCAGGCTCT
ACTGTTTACCTGTCACCCAGCTGAGCTACTGAAGCTCACTACGAATCACCCGCT
GCCCTTTAAAATAAAAGTCTTAACCAAAAGCAAGAACAACCTTATTTTGAACT
ATATTCAGCCCAATGTGACACCACAACTTTTAATCACTAACTAAAGAAAGAAA
GAACAGTGACATTATTTTAATACACATGTATTGCTTTTTATGCCGTTATCGTAA
ATCTTGACAATAACTTTGTCAGTATCGTGCTGCCAAAAATAATTTGCACACT

Locus Cm08, motif AATAG (reverse complement: CTATT)

>IH3P4PI01CQ6MB length=657 xy=1012_2481 region=1 run=R_2013_10_22_16_44_39_

AAAGAACCAAAATGTTAATGTTATTGCTGGTTAATTTACAGCATATCAAAAAA
CAACCACATGACTGTCATTGTGGAGCACATTGTAGAGGTCATCTCAGCAGCTA
ACAAATGAGTTGTATGTCTAATTACTTTTGCATGTGTTTGTTTATTGCCTTTATT
AGAAGGACATCTGGAGGAGAAGGTAGTTCATATGTCTGCTATACTATTCTGCTT
TCTGTTCTATTCTATTCTATTCTATTCTATTCTATTCTATTCTATTCTATTCTATCT
TGTAGCTTTGATGCAGTCATGTTCCCTTTAGCTGCTAACTGCCGTAAAAAGGAT
TCTGTGGTAACACTAAACATTAAGCTGCTAACAAGTGTTTTTGTGTTGCATCTA

GTAAGAGCCACAAGCGTGCCTTTTCTTGCCTTTAGAGCTGTGATTACAGTTATT
AGCTGCAGTTATTAGCTGTGGTTGTCTGTAATTAGTGCATTAAAGTGCACCTGT
GTGAACACCTGGAATCTTTTAAATTGGGCACTAAGAAGAATGTGGAATCTGAG
ACTGAACTCTCCATGTAAAACAAAACAAATAAAAAAAAGTCTATGATTCACTA
CAGCTAATTATTTGGTGAAGTCATGCTTTCCATAAATTTACATTTCTAATCACTG
AAGCAGCA

Locus Cm09, motif AGAGC

>IH3P4PI01EI8Y7 length=320 xy=1742_1441 region=1 run=R_2013_10_22_16_44_39_

GCCAGAGCCTCTGACACAGCGCTGACTGTCCACCTCCACTTACAGTCAGATTAA
AGGATGAACCCAGAGCAGAGCAGAGCAGAGCAGAGCAGAGCAGAGCAGAGG
GATGTAGGACACGAGCAGATCCACAACCAGAGAGAGAGAGACAGAGAGAGAG
AGACAGAGAGACAGAGAGAGAGAGAGAGACAGAGAGAGAGAGAAAGAGAGA
GAGACAGAGAGAGAGAGACAGAGAGAGACAGAGAGAGACAGAGAGAGAGAG
AGAGAGACAGGAAGAGAGAGAGAGAGACAGAGAGAGAGAGAAAGAGAGAGA
GAGACAGAGA

Locus Cm11, motif ATT (reverse complement: AAT)

>IH3P4PI01CFV7C length=428 xy=0884_0054 region=1 run=R_2013_10_22_16_44_39_

TACACCTGCTAAGAAGCCCTGAACTGGAATTTAATGTTAGTTTGTCCTGCACTC
GTCTCTGTTGTTTTATTCATCCATTTTTCCTCCCATAAAAATGAATAGGATGAAG
AAATTGAATTCTGTTTATTACACATCAAATAATAATAATAATAATAATAATAAT
AATAATAATAATAATAATAATAATAATAATAATAAATCAGTGTGATTCAGTGT
GATCAGTCTGCGTGTGGTTCACTGCGATTCAGTGTGCAAGTGAATTAATGTGAT
TCAGTGTGATTCAGTGTAATCAGTCTGAGTGTGATTCAGTGTGATTCAGTGTGA
TCAGTCTGAGTGTGATTCAGTGTGATTCAGTGTGATCAGTCTGAGTGTGATTCA
GTGTGATTCAGTGTGTCAGTCTGAGTGTGATTCAGTGTGATTCAGTGTAA

Locus Cm12, motif ATT

>IH3P4PI01D45N6 length=648 xy=1581_3428 region=1 run=R_2013_10_22_16_44_39_

AAGCATGCGGCTGGAGGGTTTACAGAGAACAATAAAGATCAGAGACAAAGAA
AAACAGAGGAAGGAGGAACAGTGATGAAAGTGTGCACTCTTAGGGTGGGTTG
CACCAACAACGAGTAAATTGAAGAAAGTTTAGAGCAAATCAAGGTTTTATTTT
GTTGCACCAGTTGCTTTTAAATATGGACTAACACATTTAGAGATTAAAGAATTA
ATCTAAGTTTAAAATTGAAAGCTGGATGGATTTGGCCGTATAAATAAACAGCC
CTAACATTACTCACACTAGTGGAACGTGATGTGATGATATGTGATGATATGTGG
CAATATATGATGCTATGTGATGTTTGATGAGAGGAGATGTGTGATGGTATATTA

TTATTATTATTATTATTATTATTATTATTATTATTATTATTATTATTATTATTATTAAT
CCATTCAATCATCGTCGTCAAACCCGCTTAATCCAACTAGGTGTCACAGCATCA
GGTGGAAGGCAGAATACACCCTGGACAGGCCACCACTCCATCGCAGGGACATC
GCAGGGACATTGACTTACTCACTCACACCTAGGGGTAATTTTATCATCTTTAGT
TCACCTGAACGTGCCGTCTTTGGACTGTGGGAGGAAACCGGAGCACCCGGAGG
AGACAC

Locus Cm16, motif ATT

>IH3P4PI01B6MNM length=408 xy=0778_1952 region=1 run=R_2013_10_22_16_44_39_

TTACATAAGTATTCATAAGAATACTTTACAGAAGCAGACTAAGAAGCACTGTT
CCTCTCACTCTCTGTATTATGCCCGAGTCCATGTGACTAAGGATTAGCTAGGTA
ATACACAGCTAACACTGTATGTGCATCAGTAATAATAATAATAATAATAATAA
TAATAATAATAATAATAATAATAATAATGTGTTAATGTTCTTATGCATTGTTCA
TATATAAAAGGCAATGTTATAGTTGGGTAGCAAGGTGTTGGTGCTGGTAAGCC
TTATTTTGCTCACCATATTAGCAAACGATTTGGTTTATTTAAAGCATATATTTTT
TTAAATTAGCAAAACATTTGTCAGCACAGTCAGACAATTTGGCTTATATATATT
TTTTTGAAATAACAAGTAAAAAAAAGCTTCAT

Locus Cm17, motif AAAT (reverse complement: ATTT)

>IH3P4PI01DW2SN length=622 xy=1489_3285 region=1 run=R_2013_10_22_16_44_39_

AGGTACCTATAACTATAAAGAGTTATGATAGCCAACTAAGCTAACCAACAAGC
TGCAAACAGACTTTAGCCAGACCAAAACTTAACTTATTTTAAAGATGTTATTGC
ATAGTTTTTATTTATTTATTTATTTATTTATTTATTTATTTATTTATTTATTTATAT
TTTTTTTGCAGTGGTCTCAGATATAAATGAATGACCTCTGAGCCAGTTTTGGTG
AAAAAGGTGCTCTGGTGCTCCGTTGTATTTTGGTGGAGGAGTGGGCAGGGAAG
AACAACAACAGGATTTTGGCCTCTCACCTCTAATTGGCTAACAGCACTGTGACG
TTCCCTGTTAAGACTAGAACAATATCTGTGCACTATTAGGCTAATTTGAACATA
AGAAATTATTGCTAATATTATAATTTGTGGTTTATTGCATTGTGACGGTACAAA
TCTAGGTTTTAAAAACAACCTCAAAGCAAAACCAGCCTGAAGGCAGCGCAATT
TGTAAAGTCAATGTGTTCAAAATGGTTAGAACACAGAATTAACATTGCATTGTA
TTTTGTTGGTGGTGTTCCTAATTTTTCTTTCTCAGATCTGTTCTTTGGCAAAACA
TGTAATGGTCATGTGCTATTGACGAGT

Locus Cm31, motif ATT

>IH3P4PI01BKMBM length=633 xy=0527_3184 region=1 run=R_2013_10_22_16_44_39_

ACTAGTCACACGTCACTCACTACACCCCGACTTTGTCTCTTGTGGTTAATTTTAT
GTGTTTGAGATCACTCATGTTGTCTCATGTGGGTGTGTACAATGTCCAAAGCTA
GCTAATTTAAAAGCATGAATTATTAATATTATTATTATTATTATTATTATTATTA
TTATTATTATTATTATTGCTTTTGTGGCTGACTTTATTAGCTGGTTAGCATCCAG
GCTGAGCTAGCTAGCTATCGAGAGACCTAACTGTCCAGAGACACCAACTGTAT
TTTTGGTATTTATTTTCACGCATCATCATATAAACTGGGAGAAGCAGGGACAGC
CGCTAAGGTTAGGGTACAGAAAGCCTGTTGTTCCTGGCAACATGTCAGCGGAG
TGATACAGTTTTAGTGTGAAAAATCTGTGCTGTAATCACGAATGTCAACACAGT
TAGGATGCTTCTCAACCAATCAGATTGTGATGTCGGAACTAACTCTTGTCTAAT
ATGACTAACATGACAGGTTTTGCTTTATATTTTAGTTTTTGTACAAAATTGTGTA
AAATGTGTAAAATATTTACCTTTAAGTGATATACATTTCAGAATATGACCACCT
CCTAATATTTACAAACACTGTCCATTTTATTAAGTCC

Locus Cm32, motif ATT

>IH3P4PI01ESH27 length=426 xy=1847_3073 region=1 run=R_2013_10_22_16_44_39_

GTTCTGTTTGACTCATATGGCTGAGATCAGATAAATTAGATAAATACACTGCTT
CTAGGCCTGTCCAGGGTGTATCCTGCCTTCGGCCTAATGCAGGCTGAGATAAGA
TAATTTGACAATGAACGGACACTGCTTCTAGACCATTATAGACCAAAAATAAG
CTTATATTATTATTATTATTATTATTATTATTATTATTATTATTATAGATCCATTG
ATTCGTCATGGATAGGAATGTTTGACTGAATGCAGGAGACATCAATATTCAGTC
ATTCTTTAGCCAGATTTCATGGACGGCCACAATTATACACTTAAAGGGGCTATA
TGTCCGAAATCTATTGTACTAAATCATAAAATGTCTCTCAGAGAAATAAAGAA
AACATGCTGAGCTGAAAAGCTTGTATTTCTTCTAACTGTGGTGGAGCC

Locus Cm34, motif TCGGG (reverse complement: CCCGA)

>IH3P4PI01ERH5I length=726 xy=1836_1556 region=1 run=R_2013_10_22_16_44_39_

GTGACGGGATTCGTAGATTGAGAAGAGGTGTTTCTTGCTATGCCGTCACAGAC
GGATGCCTTTGGAGACCCTAATGACCCTAAACGCTTCCATACACCTGCAAGTTC
CCCAGAAATTGAGCCTGACCCTCTCCAGGCCCGTGCACGCTCTGTCCGAACCCG
ACCCGACCCGACCCGACCCGACCCGACCCATAAACGGTCATTATGAGCCCGAT
TTAAACCCCAGGTTTTTTAACAGCAAGTAGAACGTAAACAGGCCTTTTTAACTC
CGATGTCTGATTGTGCCCGGACTCTAGTCGACTCTCCCAGCCCACAGCCTGTTA
CTCTCCACCACGTTTAGCGCTCGTACTTCACGGGACGATAGCCGGACCGTTTCA
CGCCCTCTCCGTGCGTCTAGTGTTCAAAAATAAAGGCCTATCACAGCCTCTCAC
GCACACTGTAGCGCCACACTCCTGACTCTCCTCCACACTGAGCCAGAGGTACTC
ACCGGACGTCATTCCTCTCCTCACTTCAGAAGCTCCACACGGCGCTGCAGGTTG
TGTCATGTGGACCGAGGAGCTGATGTGTGTGTGCTGCAGGCCAGAGCTGTGTG
ATTTTAACACTCTGACGTGTGACTGGTTGCTATATGGTGATTAGAGTCCGAGGG

AAGTGGTGGGAATCTGGGCCCGGCCCGNGTTGGCGTTGGGCATGGACTCGGGC
AGAGAATCTAAACTCTACCTTCTTGACA

Locus Cm35, motif ATT (reverse complement: AAT)

>IH3P4PI01DI77X length=581 xy=1332_0203 region=1 run=R_2013_10_22_16_44_39_

GAGTCTGCAATCTGATGTATGATGACATCCAAACAGCTGTCTGGAGATAGAAA
TACACATTCACCAAGGACTTTATTAGGAACACCTGTACACCTACTCATTTATGC
AAGTATATAATCAGCAAATCATACGGCTGGAGTGCAGTGCATAAAGTCATGTA
CATACAGAACAGCAGTTTCAGGTAATGCTTAATAGCCATTAGCAAAAGTGTGA
CCTGGCATGCTTGATGGTGCCAAGCAGATTGGTTGAAGTATTTCTGTATCTGCT
GATCTCTTGGGAGTTTCACGAACCCCTGTAAATAATAATAATAATAATAATAAT
AATAATAATAATAATAATAAAACATCCAGTAAGCAACAATCCTGTGATGGAAA
CACTTTATTAATAAGAGAAGCCAACAGAGAATGGCCAGACTTGTTCAAGCTGT
TAGAAGAACTAGAGCATCATAGAAAACATAACATAACAAGGCTTGAGGTGAAT
GGGATACAACAGCAACACTACATAATGTTCCTGAGGCTGCAGTGGGCACAAGC
TCACCAGAAATAAACAGCTGAAGACTGCAAAAAACATAATCTGGTCAG

Locus Cm36, motif ATT (reverse complement: AAT)

>IH3P4PI01EXS6L length=583 xy=1908_0875 region=1 run=R_2013_10_22_16_44_39_

AAATGTTCAAGATTTTGCAATTTTATTCATTTATTAATACTTTTTTGTCAACTGG
AAGGTTCATTAAGACCATTATATTCACCCTCAATGTTAGTTAGCAAATACTAAA
AGCAAACTTAAGTTAGCTGATAGCTACACACGTTATTTGTTATTTGTTTTGATA
ATCAGTAGATTATTTGACTATGAAATAGTCTTGTTGCAGTCCTGTCTTGCAGAC
ATCATTGTTGGCCCCGGAATAATAATAATAATAATAATAATAATAATAATAAT
AATAATCCTCATTAATTTGGACTCCTAATAATCCGTCTTTTTTCTGGCACGTTAG
ACTTTATTGAGCTGGATTCGTGCTTGGACCCCTTGTATTGTTGTCTTCCTGGATG
TTAAACTTAGCAAATGAACAGTCATTTGTGCATCATCGCCTGTAGAACATGCAA
ACGTATTTCTCCCTAAAAAAAATCATCTTATTTGACTTTGATGTTGGCTCCTTTT
AATCGTGTCTTTGTAGTAACCGTTGCTCTTATTAGCTTCTGTAAAGAATAGAGC
ATAAGAGCAAGAACCCAGTGAAAATGTCCTCATTGAGTGA

Locus Cm38, motif ATCT (reverse complement: AGAT)

>IH3P4PI01C44GU length=304 xy=1171_1612 region=1 run=R_2013_10_22_16_44_39_

GCATCATGCATAAAAGTGCCCCAGATTTTGTACTTAACACTTCCTCTTGTCTGCT
TAAGAATAGATAGATAGATAGATAGATAGATAGATAGATAGATAGATAGATA
GATAGATAGATAGATAGATAGATAGATAGATAGATAGATAGATAGATAGATA
GATAGATCAGGACATGACTCAGGTACCCTTGAGCAAGGCATCTAAGCTCCAAC

TGCTCGCCGGGCGCAGTGTGTGTGTGTGTGTGTGTGTGTATAGTGTTTTACTGC
ATGGATGGGTTAATTAACTCTTGTATGCAAAAAAAAAG

Locus Cm39, motif TTC

>IH3P4PI01DIR3Z length=764 xy=1326_3901 region=1 run=R_2013_10_22_16_44_39_

AGCCCTCACAGGCTGTTTAACCAACAACATGTTTATAAAGTGAGAGTGGAATC
AGTCAATTACAGTGTGAGTTTCAGTGGGTGGGATTAATGTTGAGAGAAAAACG
TCGCTTTAGGCTTCCTGCAAGGCCAAAATATGTCACTGATTGCGGGCATGAGCA
GTGAGACTACGTTAGCTAGTTTAGCTTATTAAGTCTGTTCATGCGCACTTCCAC
ATTAACTACGTAGTCACTGTTTGATACAGCTAACTTTATACCATCTTTAGTGATT
GCGCAATACTTATGTTATGCAATTTATATTCTTCTTCTTCTTCTTCTTCTTCTTCT
TCTTCTTCTTCTTCTTGGCACACAATTTTTGTCCACTACTTAGTCTTGTAGTTTTT
GAGATATCGACACTGCATTTCTGCATAATTCAATTTTTTTCCACAGAGAATTA
ATAAGACTGAAAGGTGCATGCCCACCCCTGAGCACTAATTCACCCTTGGCTTGA
TCCGCACTCATCCTATTTTACTCCCTTAAAGACCTGTTTATACTTCAGGCCTTAA
CTAAAACTAGATAAAACTCAACTAGNTATCGACATCGTTCCACTTCCAGATTGT
TCGGCCTGATCGGGATTTATGTGCTTGTATACAGCCTTTTGATCGGACTCCCGG
ATGCCGTGTAATTCCGGATTTTATCTGATTTTTATCTGATCTGATCTGATTTTCC
CATAGAGAATGAAGGGATGGAACGTGTATGTCTACCTACCAGAGATGCTCACA
AGTC

Locus Cm40, motif AGTG (reverse complement: CACT)

>IH3P4PI01BS2P9 length=487 xy=0624_0383 region=1 run=R_2013_10_22_16_44_39_

GAAGCATTTGGATTTGGGACACTTAACCTGCTGTAACCTCTTATTTCTTAACTAT
TGTCTACACTGCCTGCATAATTTTTTCGTGGTTCTGCTCTGTTTCTGCTCTGTTTC
ATCTACATTCTCTGTGTTTCCATGAAGGATATGAAGGATATATGAAGAAAAAAT
TGAAGGAAATGATTAGACAAAAAGACTCCATCTATATCTGTATTAAATGTTGA
GCATGAATGAGTCGGTACTTACAATATCCAGAATATGTCCAGAAAAGTGTACA
TATCATTTATTTAAATGATGATAAAACATAATTTAGCCCACCACTAATCACTCA
CTCACTCACTCACTCACTCACTCACTCACTCACTCACTCACTCACTCACTCACTC
ACTCACTCACTCACTCACTCACTCACTCACTCACTCATCTTCTAACCCTATCCCA
GTGTTCATAGGGCTCATAGGTCCCATTTTACTGCTTAATTAAGCCTGGAAGC

Locus Cm41, motif ATCT (reverse complement: AGAT)

>IH3P4PI01D0P3R length=329 xy=1531_1429 region=1 run=R_2013_10_22_16_44_39_

GGTGGTCCTGTGTGGGGGTCCGGACTATTAAAGTACAGTGTGAAAGGGACAAA
TATATAAAGTATGCAGAGAATAGATAGATAGATAGATAGATAGATAGATAGAT

AGATAGATAGATAGATAGATAGATAGATAGATAGATAGATAGATAGATAGAA
AGATGTGGATGCACTGCAGTAGCATGCCTCCAGATACCTGTGACAGCTTACTA
GACCCTTATTAAGCCACTCCTTGTCCATCTAGATGCTGTCCGTGCTGCACACGG
TGGTTGCTCTGGATATTAGCCGGTGGACTGGGTATCACTGCCCACAAAAACTTT
GTATCTCTTT

Locus Cm42, motif AAC

>IH3P4PI01EBH28 length=653 xy=1654_0450 region=1 run=R_2013_10_22_16_44_39_

GGCTAATGCTGATCACCTTTGATATCTGATTCCTGGAATATGCTGATGAAGGTA
ATCTGGGTCAGCAGGAATCACTTATCACCCCTCAGTTACTGTCACGTTCACTCT
TATTATCACTCCGCCTCTTACTAACACACTCCTCCAGCTCGTTAGACACTTTCTT
AATGCTTCAATAAATCTTCAATTAATAATCTTAATTAACACTTCTTAACCAGGA
GCTCTAGATCAGTCACCGACGACAACAACAACAACAACAACAACAACAACAA
CAACAACAACCACACCATACAGTCCTCATACAGGCTGAGGGTCTTCCACCAAG
ACCAAGCCACATGTCACACGGCTGCAAGGACCCCTAAATCCCCAGACCTGAGC
CCAACTGAGCATCTGTGGGACCACCTCAATTATTGTGTTCACTCTATGGAGCCT
CCTTCACACCCCTCTAGAAGCTTTGGGATGCACTGCTGTCAGCATGGCTCCAGA
AACCCGAGACAACCTACCAAGACCTTCATGAGTCACGCCCAGCCCATTCAGCT
GCTGCACACAGATACAACTGTGTATGTATACTGTTTATCATGGATCTCTAGACT
AGAACTCCATTTATACACTCAGTAGAGTCGTGTAGGTTCACTCGTGGTTCATAT
ATACACTCA

Locus Cm44, motif ATAGT (reverse complement: ACTAT)

>IH3P4PI01EJRCG length=312 xy=1748_0670 region=1 run=R_2013_10_22_16_44_39_

ATACTTATACTATACTACACTACACTACACTGTGTAATATTATTTTAAGTAAAG
GTTCCTAAATGGTTCTTAGATTTGCATTACATACATTACATCATATTACACTTAT
ACAAGGTGCCAGCTATTATCAGTTATCATTTAAAAGATTTCATATTACTATATA
CTATATTATATACTGTGTACTACACTATACCATACTATTGTATACTATACTATAC
TATACTATACTATACTATACTATGTCTAAATCTATTTAAAGTAAAGTGAAAAAG
GAAATGTGCAGGATGCTCACAGGCTCTTCAACAGTAGGTG

Locus Cm45, motif ATT

>IH3P4PI01DULWP length=440 xy=1461_2775 region=1 run=R_2013_10_22_16_44_39_

AAATGAATATAAGATCAGTGCAAACAAGTATAATTCCTTTGATATAAATATTA
GTTAATTATCTTAACATTGAATTAAAATGAGCTTAAGTGTATTTAATAAGGCTA
ATCTTCTTTCAAGCTTAAAATGTTTATTTAAAATATGCTAATGATTTGCCAGTGT
AATGAGAGGATCTGTCTGACTAAACTCAAAACGAGTAAAATGTTTCTTGAAAT

TGTTTAAATATCTTAAAACAATTTAACNACATATTAACTACATATTGACTTGCC
ATTTACCAAGATATTAGTTTCTGTAGTGCATGTGTTTGTGGTATTATTTAAAATA
TATTATTATTATTATTATTATTATTATTATTATTATTATATATATTATTAAGAATT
TTGTTTTCTATTTATTCGGCTGATTATAAGATTAAAACCATGTAAAATTGTAAC
ATCGCT

Locus Cm46, motif ATT

>IH3P4PI01B4EKY length=232 xy=0753_0576 region=1 run=R_2013_10_22_16_44_39_

ATAATGAATAATTATTAGGGTAGCACAGTGGCAGTGGTGTCTTAGATTTCCTTA
GATTGGGCCCAAAAATGGAGCCAACTTAGCCGTGCACATTCTGCCAACATTAA
CTGTCATTATGAGCATTATTATTATTATTATTATTATTATTATTATTATTATTATT
ACCTGTATATATAGTAGTATATATAGTAGGTAGTTTGGGTGATACAGTTGCTGTAT
CACTGTAACTGATAG

Locus Cm47, motif ATT (reverse complement: AAT)

>IH3P4PI01D1EFW length=348 xy=1539_0202 region=1 run=R_2013_10_22_16_44_39_

GTACAGCCGCTACGTACCCGGATGGAGGAGTTTCCAGACACTATAGTTATGTG
CTTCTGCATAGTCATCTGCAGCATAGGAGTCTAACGGAGGGGAGCAAGCTTAT
CAAGGAGATAATAATAATAATAATAATAATAATAATAATAATAATAATAATAA
ATAAAAAATGTTACAAATAATAATCGTTAAAATTAGACATTTATAATTATATAG
TCAGCGGCCATTTTATTAGGTACTTGTTTGTACACCTTCTGCTCATTTTTACCTT
TTAGGTGCACTTTGTAGTTCTGCACTTACAGACTGTAGCAGTTCTGTTTCTCTGT
ATACTTTATTACCCTGTTGCTTTAA

Locus Cm48, motif ATT

>IH3P4PI01DAEJC length=451 xy=1231_2182 region=1 run=R_2013_10_22_16_44_39_

GAATGTCTGATACCTGCTAGTTTAATAGTATTTTAATGATGTTCTGGCTCTAAA
ATCATCGTGTATAACTCGTAGGGATGGGTACAAACGAAGGGTATCGAAGGGTA
CAAACGAACCAGAGTGCTGGAGTGAAAACACCCTAAGTTTATACTGTTATTTA
GACGCCTTCGCTCAGCATGTTACATATGCAGAACATTTCTATATGATGTTAATA
ATAACAATAATAATAATAATAATAATAATAATAATAATAATAATAATAATGGG
CAGCATGGTGGCGCACGGTAAACCACACCTGCACATATAAATAAGTTGTATCT
TTAATAAGAGCTTCTCAGCAGCTGATCTGCAGGCTCTGTGTTTGATGTGTACTT
ACGAGGCAGAGGCAGGAGGTGGTTGTGGTGTGGTTGTCTGTGTTGTGAGGTGG
TGATAGTGTGTGTTGTGAGGTGGT

Locus Cm49, motif ATT

>IH3P4PI01AV1DR length=233 xy=0247_3181 region=1 run=R_2013_10_22_16_44_39_

TGATCTTTCCTCAGAACTGTGTTCATAAAGAGGTTACCTGCTCCACTGTGACAT
GTTGCTCTGTACCACTCACCGGAGTCATTGTTGAACACATTATTATTATTATTAT
TATTATTATTATTATTATTATTTATTTCAACCCAACAAATGACCCACACAGTCAT
TCTGACAGACTGTATTACCCTACAGGAAGTGTAGGGGCGATATATGGACCTAC
GTGGTCATTCTGATGG
