# Supplementary figures and images for: Microsatellite loci development and population genetics in Neotropical fish Curimata mivartii (Characiformes: Curimatidae)
Source: PeerJ. 2018 Nov 13;6:e5959. doi: 10.7717/peerj.5959 (PMC6238776; doi:10.7717/peerj.5959)

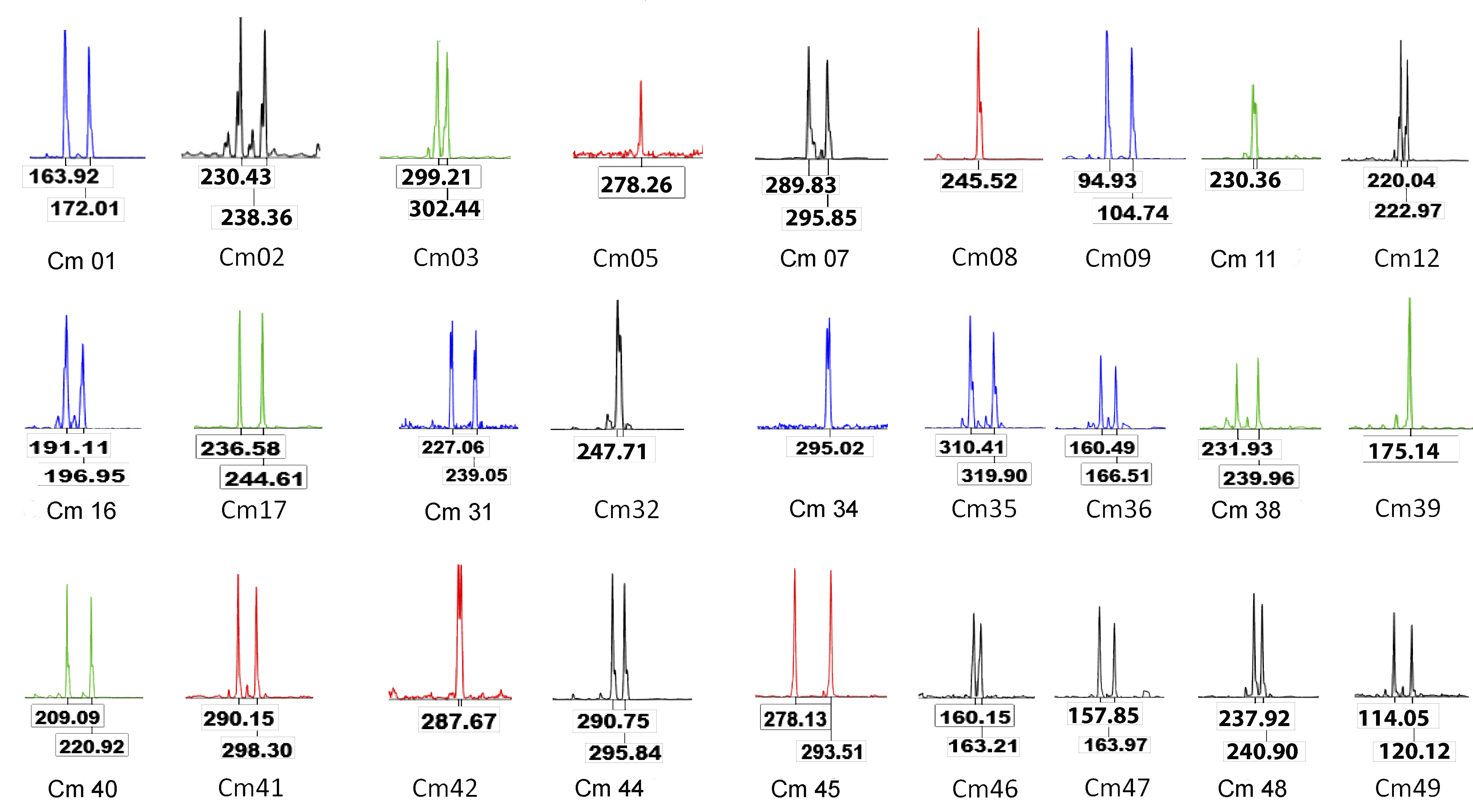

Supplement: Supplemental Information 5 [file peerj-06-5959-s005.png]
